# Bladder cancer biomarkers: current approaches and future directions

**DOI:** 10.3389/fonc.2024.1453278

**Published:** 2024-11-29

**Authors:** Melika Ahangar, Frouzandeh Mahjoubi, Seyed Javad Mowla

**Affiliations:** ^1^ Department of Clinical Genetics, National Institute of Genetic Engineering and Biotechnology (NIGEB), Tehran, Iran; ^2^ Department of Molecular Genetics, Faculty of Biological Sciences, Tarbiat Modares University, Tehran, Iran

**Keywords:** biomarkers, diagnostic strategies, prognosis strategies, monitoring strategies, FDA approved molecular biomarkers

## Abstract

Bladder cancer is a significant health concern worldwide, necessitating effective diagnostic and monitoring strategies. Biomarkers play a crucial role in the early detection, prognosis, and treatment of this disease. This review explores the current landscape of bladder cancer biomarkers, including FDA-approved molecular biomarkers and emerging ones. FDA-approved molecular biomarkers, such as BTA stat, BTA TRAK, and NMP22, have been instrumental in diagnosing and monitoring bladder cancer. These biomarkers are derived from urinary samples and are particularly useful due to their sensitivity and specificity. As we move forward, we should continue to seek ways to optimize our processes and outcomes, these markers remain seriously challenged in the detection of early bladder cancer due to their limited sensitivity and specificity. For instance, sensitivities of BTA stat in bladder tumor detection have varied between 40-72%, while its specificities vary from 29-96%. In the same way, 70% sensitivity and 80% specificity have been recorded for BTA TRAK, while 11-85.7% sensitivity and 77-100% specificity have been documented for NMP22 BladderChek. The given variations, especially the low sensitivity in the diagnosis of bladder cancer at an early stage call for the invention of better diagnostic systems. Moreover, different sample collection and handling procedures applied in different laboratories further contribute to inconsistent results obtained. Extracellular vesicles (EVs) and exosomes, which carry a vast number of proteins, are being considered as potential biomarkers. Although these markers show promise, challenges remain due to non-standardized isolation techniques and lack of reproducibility across studies. Moreover, the discovery of new potential biomarkers is ongoing. For instance, the UBC^®^ Rapid test and UBC ELISA kit, the XPERT BC Monitor, BC UroMark, TaqMan^®^ Arrays, Soluble FAS (sFAS), Bladder tumor fibronectin (BTF), and IGF2 and MAGE-A3 are among the newest biomarkers under investigation. In conclusion, while bladder cancer biomarkers have shown great promise, more research is needed to standardize the testing procedures and validate these biomarkers in a clinical setting. This will pave the way for more accurate and efficient diagnosis and monitoring of bladder cancer, ultimately improving patient outcomes.

## 1 Introduction

In order to locate and examine papers about bladder cancer biomarkers(Biology has several tools that can help understand the metabolic situation of a living organism better. Among them is a biomarker which is a specific type of biological marker or a measurable indicator. Many definitions describe a biomarker as a biologically derived characteristic that can be objectively measured and evaluated as an indicator of normal biological processes, pathogenic processes, or pharmacologic responses to a therapeutic intervention. Following the analysis, biomarkers may be detected in various biological fluids or tissues, allowing them to be utilized for disease diagnosis, treatment monitoring, risk assessment, or drug effect assessment. The types of these markers include though are not limited to molecules, cells, and proteins. However global clinical practice considers them as necessary tools while targeting specific clinical endpoints or biological changes(CBPI). In this regard, it should be emphasized that the biomarkers reflect the characteristics of particular tissues or organs but not diseases), we conducted an extensive search of the literature (1, 2). The keywords for the search were bladder cancer, bladder cancer biomarkers, biomarkers, Diagnostic Strategies for bladder cancer, Monitoring Strategies for bladder cancer, prognosis strategies for bladder cancer, and FDA-approved Molecular Biomarkers in bladder cancer (3, 4). To focus on pertinent studies, we used advanced search filters and several databases, such as PubMed, Scopus, and Web of Science. Our search approach was created to include both current developments and body of knowledge, to provide a comprehensive and fair picture of the state of the field’s study. To guarantee the caliber and applicability of the included studies, we strictly regulated our selection criteria. Peer-reviewed English-language articles with a strong impact factor were our main emphasis. Furthermore, original data and analysis were given precedence over reviews and meta-analyses in our ranking of studies. To present a thorough picture of the methods being used today to find and validate bladder cancer biomarkers, we also tried to incorporate a range of study formats, including case reports, observational studies, and experimental investigations. Following the identification of promising research by our preliminary search, we conducted a comprehensive screening procedure to ascertain their suitability for inclusion in our review. This required reviewing the complete texts and abstracts of the studies that were found, determining how relevant they were to our subject, and analyzing the methodological soundness of each one. To make sure that each study wasn’t just repeating prior research without offering any fresh perspectives, we also looked through its references. We were able to preserve the integrity of our review and make sure it appropriately reflects the current status of research on bladder cancer biomarkers thanks to our stringent screening procedure (5, 6).

## 2 Bladder cancer pathogenesis and subtypes

Bladder cancer is typically characterized by the rapid growth of cells in the bladder. This balloon-shaped hollow organ located in the lower abdomen stores urine. It is constituted by a muscular wall that can expand to retain urine from the kidneys and contract to expel it from the body. On either side of the back, above the waistline, there are two kidneys. The bladder and kidneys work together to eliminate waste through urine and cleanse the rest of the body (7).

**Figure 1:**
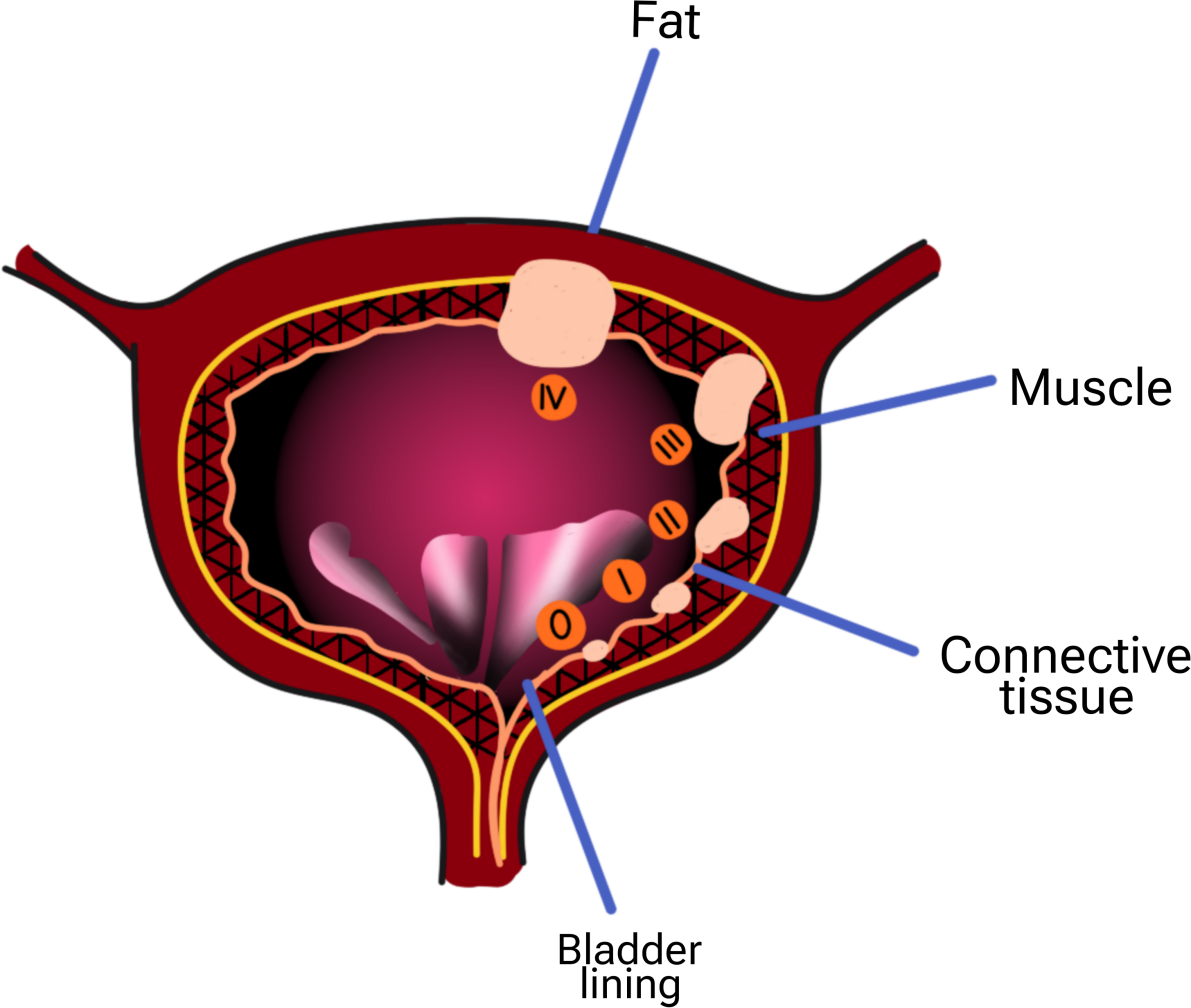
Stages of bladder cancer) 0:The cancer has grown only in the center of the bladder and has not spread into the tissue or muscle of the bladder wall or spread to your lymph nodes or other organs. i: The cancer has grown into the wall of your bladder, but has not involved the bladder wall muscle. ii: The cancer has grown throughout the connective tissue in the bladder and into your bladder muscle. iii: The cancer is now in the layers of fatty tissue surrounding your bladder. This cancerous tissue may be in the prostate, uterus, or vagina, but it has not reached the nearby lymph nodes or distant organs. This step may include any of the following: Cancer has spread from the bladder to your pelvic wall or abdomen, but not to nearby lymph nodes or distant organs. Cancer has spread to nearby lymph nodes but not to distant organs. The cancer has already spread to your lymph nodes or distant organs, such as your bones, liver, and lungs.

As mentioned in **Table 1**, there are different types of bladder cancer which have been introduced with detailed explanations. In addition to dividing the types of bladder cancer, it has been introduced the different stages of bladder cancer in **Figure 1**. Urothelial carcinoma is another name for transitional cell carcinoma, which is also known as urothelial neoplasm; it is one type of bladder cancer among several other types that exist. Urothelial carcinoma originates from urothelial cells lining inside the surface of the urinary bladder (8); other types include adenocarcinoma, squamous cell carcinoma, small cell carcinoma, and sarcoma respectively; each has its unique treatment approaches depending on its nature (9, 10). For instance, surgical approach chemotherapy and radiation therapy are mostly used for managing urothelial carcinoma, an operation called cystectomy may be conducted to remove all or part of your bladder; this is done along with removing other surrounding organs such as urethra (a tube leading out from your body) (11). Conversely, small cell carcinoma often does not respond to surgery or radiation therapy and thus chemotherapy frequently becomes its mainstay treatment (12).

**Table 1:** Based on the information from the search results, this table shows different types of bladder cancer along with their details.

Type of Bladder Cancer	Details	Reference	Diagnostic and PrognosticBiomarkers
Urothelial (Transitional Cell) Carcinoma	This is the most common type of bladder cancer, making up about 95% of cases. The cancer cells of this type look like the urothelial cells lining the inside of the bladder. It can also affect the kidneys and the ureters that connect the kidneys to the bladder.	(16)	FGFR3mutations (diagnostic), TP53 and RB1 alterations (prognostic)
Papillary Carcinoma	This subtype grows out from the inner surface of the bladder towards the hollow center in finger-like projections. It is often referred to as noninvasive papillary cancer, meaning it doesn’t grow into the deeper layers of the bladder wall.	(17)	FGFR3mutations (diagnostic), TERT promoter mutations (prognostic)
Flat Carcinomas	This type of Transitional Cell Carcinoma does not grow out of the urothelium towards the center of the bladder. Instead, flat carcinomas remain on the surface of the bladder wall. If a flat carcinoma is confined to the urothelium, it is called noninvasive flat carcinoma or flat carcinoma *in situ*.	(16)	TP53 alterations (diagnostic), MDM2amplification (prognostic)
Squamous Cell Carcinoma	This accounts for about 1% to 2% of bladder cancers diagnosed in the United States. Squamous cells look similar to the flat cells on the surface of the skin. Almost all squamous cell carcinomas of the bladder are invasive.	(18)	PIK3CAmutations (diagnostic), overexpression of EGFR (prognostic)
Adenocarcinoma	This type of bladder cancer closely resembles the gland-forming cells seen in colon cancers and accounts for about 1% of bladder cancers in the United States.	(19)	CTNNB1mutations (diagnostic), high expression of HER2 (prognostic)
Small-Cell Carcinoma	This is extremely rare, accounting for fewer than 1% of all bladder cancers diagnosed in the United States. This type of bladder cancer begins in neuroendocrine cells, which are similar to nerves.	(20)	ChromograninA, synaptophysin, and NCAM (CD56) (diagnostic); high Ki-67 index (prognostic)
Sarcoma	This is another very rare type of bladder cancer that begins in the muscle layer of the bladder wall.	(21)	Desmin, MYOD1, and muscle-specific actin (diagnostic),MIB-1 proliferation index (prognostic)

Note that the exact details of each type can vary depending on the specific case and the individual patient’s overall health status.

**Figure 2:**
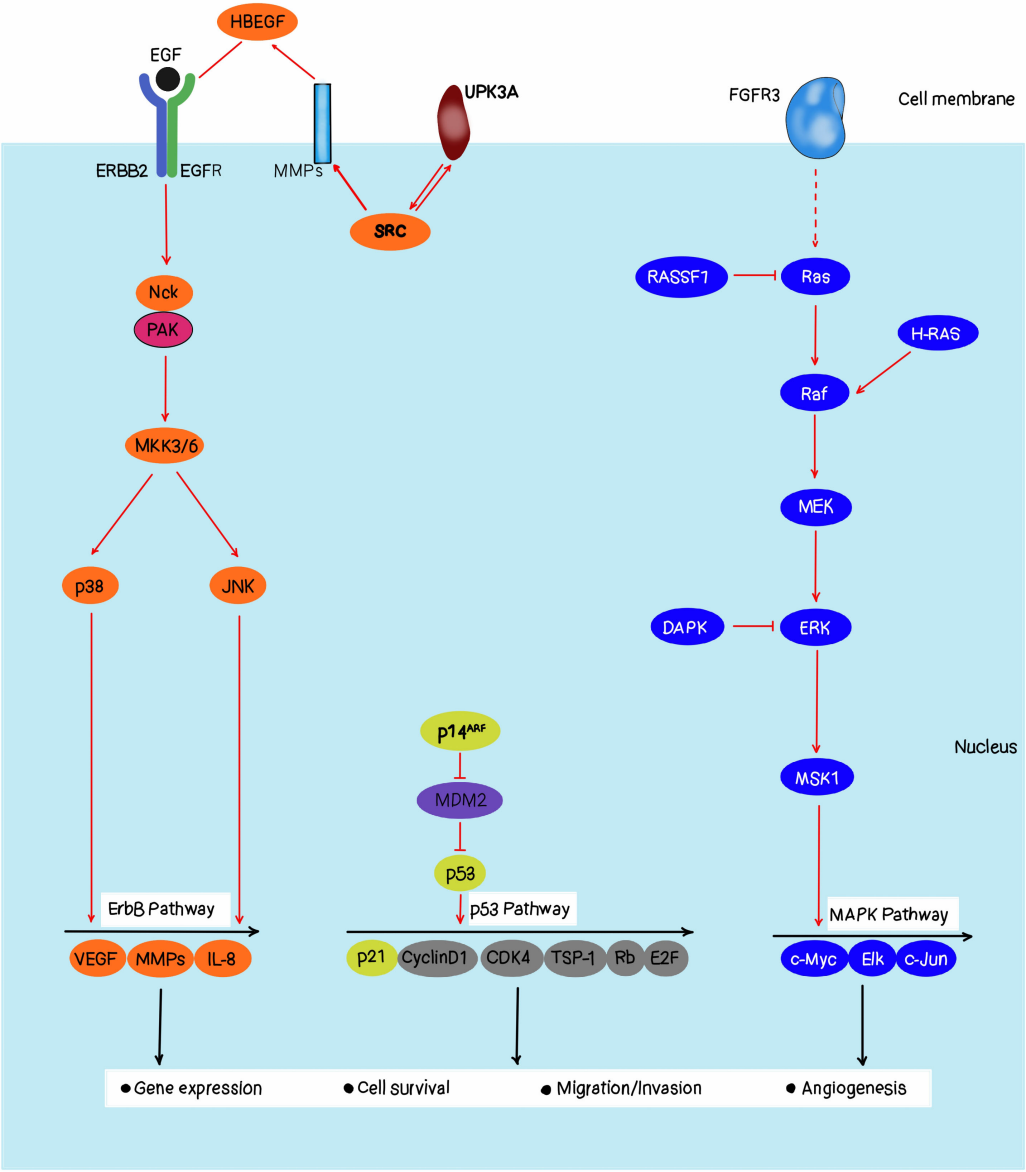
Bladder cancer pathways.

**Figure 2** is the Ras-RAF-MEK-ERK signaling pathway that plays as essential role in coordinating cell proliferation, differentiation, and survival according to extracellular stimuli. RAS is the most frequently mutated oncogene in bladder cancer, with KRAS, HRAS, and NRAS involved (13). It was found that approximately 36.4% of high-grade muscle-invasive bladder carcinoma had a mutation of the MAPK (mitogen-activated protein kinase) pathway, which is the second activating pathway than TGF-beta signaling remained after partition by genome-based clustering, highlighting the MAPK pathway may be an opportunity therapeutic target for this devastating disease. In the past few years, the mechanisms that integrate the Ras-RAF-MEK-ERK pathway into the bladder cancer phenotypes have started to accumulate, including a subset of bladder tumors with focal amplifications of RAF1 gene, and found that these amplifications drive activation of the canonical MAPK pathway (14). This subgroup of mutant RAF1 forms accounts for nearly 20% of urothelial tumors as they are dependent on RAF function for their growth. The evidence is also available that TP63+ bladder cancers, which all bear either HRAS or NRAS mutations, are particularly sensitive to RAF1 pathway inhibition. Thus, it is suggested that these cancer cells are dependent on this pathway for their growth, so can be preferentially targeted by compounds that inhibit the MAPK pathway. This mechanistic understanding has significantly improved our understanding of bladder tumor biology and has presented us with a critical opportunity for the development and testing of MAPK pathway inhibition for bladder cancer therapy (15).

### 2.1 Molecular basis of bladder cancer

There are numerous biases in the area of genetic expression studies related to bladder cancer research. One source of bias is the highly dynamic expression of the transcriptomes, which include various RNA species and complexities of function at the gene and isoform levels. This essentially creates complications that may result in the identification of discrepancies in gene signatures through various cohorts. Moreover, the essentially heterogeneous transcriptome environment in each of the patients adds more variability, which is another reason for the inconsistencies that may arise in gene signatures when used on heterogeneous tissue samples (22).

Differences arising from the sequencing technology, data analysis algorithms, and analysis software are contributing factors that generate bias in genetic expression studies. Furthermore, in single-cell RNA-seq differential expression analysis, the possibility of false discovery is a major challenge for applying gene signatures in clinical scenarios. Such challenges make it clear that they should be dealt with through standardization of protocols of work throughout the experimental level, combining suitable experimental design with advanced analysis practices for the improvement of reproducibility (23). Future directions include multi-omics data integration, machine learning methods, open science endeavors, and collaborative acceptance of developing more robust and repeatable gene signatures to aid in the diagnosis and treatment of bladder cancer. By addressing these biases within future design methodologies, top scientists are in a better position to translate genetic expression ramifications into effective clinical guidelines for the treatment of patients with bladder cancer (24).

Bladder cancer is a complex molecular disease and multifaceted, with genetic, epigenetic, and environmental factors playing roles (25, 26). It is crucial to comprehend these constituents to develop effective diagnostic and therapeutic strategies. One of the primary molecular mechanisms underlying bladder cancer includes Ras-RAF-MEK-ERK pathway activation. Consequently, dysregulation of this pathway is common in bladder cancer which leads to uncontrollable growth and proliferation of cells. Most of the time, bladder cancers have mutations in RAS gene which codes for Ras protein causing its activation along this pathway (27, 28). Such an activation may cause over-expression of some oncogenes like EGFR thereby further promoting carcinogenesis. Epigenetic modifications are also known to play a significant role in bladder cancer development (29, 30). Epigenetic modifications such as DNA methylation or histone modification can change gene expression without altering the underlying DNA sequence itself (25, 31). These changes can lead to tumor suppressor genes’ silencing and oncogenes’ activation in bladder cancer for example TP53 gene well known as tumor suppressor is methylated on its DNA resulting in loss of function hence causing bladder cancer formation (32). Also, the development of bladder cancer is affected by environmental factors such as exposure to some chemicals, and tobacco smoke. For instance; tobacco smoke has been associated with an increased risk of bladder cancer since it contains some chemicals in it which are carcinogenic (28). In addition to that, exposure to certain industrial solvents such as aromatic amines can also increase the risks of getting cancer of the kidney (33). Bladder cancer’s molecular basis entails a multifaceted interplay between genetic epigenetic and environmental factors. This understanding is important to come up with therapeutic approaches that target these elements. Moreover, more investigations into these areas are needed to improve the prognosis and quality of life in patients suffering from bladder cancer (34).

Mutations in genes like RAS, particularly H-Ras, N-Ras, and K-Ras, play a significant role in bladder cancer progression:

Mutation frequency: H-Ras mutations are present in 0-12% or up to 30% of the cases of bladder carcinomas as analyzed. Other molecular analyses that have been conducted recently have demonstrated that at least one of the three Ras canonical proteins is overexpressed in 77% of bladder tumors compared to the adjacent malignant tissue (7, 35).

Mechanism of action: Ras genes are involved in cell signaling and oncogenic mutations distort the fine balance and future path of cell signaling thus resulting in tumor formation. These mutations cause the Ras proteins to become constantly switched on by the pathway, so stimulating cell growth and division and leading to the formation of tumors (36).

Specific mutations: There is a significantly higher incidence of G12V mutation in the H-Ras protein among bladder carcinoma samples (G12V = 60% of the total number of affected H-Ras samples identified), while G12D and Q61R were at 8% and 7% respectively. These mutations result in Ras proteins always being active, this is necessary for a cancer cell’s survival and ability to divide (37).

Role in bladder cancer progression: The molecular analysis of H- Ras has been documented to be involved as primary changes during the initial stages of bladder cancer. They are mainly associated with benign tumors although very rare do transform into higher stages of malignancy. On the other hand, it has been found that a single nucleotide polymorphism (81T>C) was correlated with significantly increased odds of the development of bladder carcinomas particularly advanced and more malignant ones (38, 39).

Therapeutic implications: Inhibition of the Ras-RAF-MEK-ERK pathway leads to a new trend in cancer treatment. A range of inhibitors targeting this pathway have been synthesized and exist either in current clinical practice or are under investigation. Other recent developments include the U.S. FDA’s recent approval of Sotorasib as an orally bioavailable selective KRAS G12C inhibitor, following the dream of targeting previously considered “undruggable” targets (40).

Challenges and future directions: Despite this, there have been some problems in the management of cancer through the development of resistance to RAF and MEK inhibitors. Despite these promising results, the current study highlighted several limitations including mutational subgroup specificity and drug resistance owing to tumor genomic variability. To better understand the specifics of Ras signaling involvement in these types of cancer, further examination is still needed, specifically involving the acting types of cancer like acute myeloid leukemia and acute lymphoblastic leukemia (41).

Lastly, bladder cancer is augmented through RAS gene mutations that work through the Ras-RAF-MEK-ERK path signaling leading to cell proliferation. Knowledge about these mechanisms is important for the design of targeted therapies that would be beneficial for patients diagnosed with bladder cancer (40).

## 3 Conventional bladder cancer detection

There are different ways of detecting bladder cancer. At first, a comprehensive discussion with a healthcare provider is done which will be based on the symptoms experienced by the patient and risk factors that he or she could have contracted bladder cancer. Physical examinations are conducted to rule out infections which are more common than bladder cancer as well as urinalysis and urine culture (42). One of the main ways of diagnosing this disease is cystoscopy. During cystoscopy, a urologist utilizes a lighted tubular instrument known as the cystoscope to visualize the inside of the urethra and urinary bladder. Biopsy specimens can be obtained immediately if there is any suspicion of a tumor or other abnormal growths in the bladder. The pathologist would then look at the biopsy under a microscope to see whether it contains cancer cells (42, 43). Other tests include urinary cytopathology, in which urine samples are tested for signs of malignancy or precancerous conditions using microscopy, and urine markers for tumors that detect specific substances released by malignant cells present in the urinary system (44). Bladder cancer may also be diagnosed by imaging techniques such as computed tomography (CT) scans or intravenous pyelogram (IVP). A CT scan uses X-rays to make a detailed images of the body to provide information about cancerous formations within the urinary system which is essentially the bladder (45). Alternatively, one can look for bladder cancer using an ultrasound. It makes use of sound waves to capture images inside the body, these images help in determining the size of bladder cancer and whether it has spread to nearby parts (46, 47).

### 3.1 Biomarker

Biomarkers are chemicals in the human system that can signal a disease or disorder. In respect to bladder cancer, biomarkers can be traced in different body fluids and tissues. These biomarkers are proteins, nucleic acids, or cells that can give crucial information about the presence of cancer (48).

Three major biomarkers used in diagnosing and treating bladder cancer include Bladder Tumor Antigen (BTA) STAT, BTA TRAK, and Nuclear Matrix Protein 22 (NMP22) (1, 49). These tests rely on detecting complement factor H-related protein found in the patient’s urine, which is produced naturally by our bodies as a means of protecting cells from being activated by complement. The structure of this protein is very similar to that of the complement factor-H-related protein made by cells affected with bladder cancer (50, 51). Point-of-care “immunochromatographic assay” that uses five drops of urine and gives results within 5 minutes is done using the BTA STAT test (48). On the contrary, standard quantitative ELISA measurement quantifies antigen levels as done by the BTA TRAK test. Both tests have been approved by the FDA for surveillance alongside cystoscopy examination (1, 42).

Nevertheless, they are not without their flaws. BTA testing has been found to have a diverse range of sensitivity and specificity (52). False positives have also been seen in hematuria, urolithiasis, inflammation, recent instrumentation, other genitourinary malignancies, and intravesical BCG^1^ therapy so that the specificity of these tests can be significantly reduced (53).

Alternatively, NMP22 is a nuclear matrix protein that is released into urine as bladder cancer progresses. It is widely used for the detection of bladder cancer including its recurrence after transurethral resection (TUR) (54). There was a study conducted on 179 patients which showed that the NMP22 test had a sensitivity of 74% and specificity of 55%. Like BTA tests, NMP22 cannot substitute cystoscopy and should not be considered a routine part of surveillance after TUR in superficial bladder cancer patients (55).

Thus, while useful tools for managing bladder cancer include BTA STAT, BTA TRAK, and NMP22 alone they might have limitations such as sensitivity or specificity and they can produce false positive results; therefore other diagnostic methods such as cystoscopy must be used in conjunction with them (54, 56).

#### 3.2.1 Commonly recognized groups of biomarkers in bladder cancer

Protein biomarkers: These come from the proteins that bladder cancer cells make. Mass spectrometry, liquid biopsy, and immunohistochemical methods can all be used to find protein biomarkers. A few examples are PDGFRB, EGFR, and ERBB2.

PDGFRB (Platelet-Derived Growth Factor Receptor Beta):

PDGFRB abnormalities have been reported in some cancers such as colorectal cancer (CRC).

The relationship that PDGFR mutations bear to EGFR inhibitor resistance is unclear, but such a connection might exist. A multi-kinase inhibitor selective for PDGFR indicated activity in CRC patients with the worst response to panitumumab/cetuximab treatment (57).

EGFR (Epidermal Growth Factor Receptor):

EGFR expression has been noted to contribute to ovarian cancer progression and prognosis when amplified and/or over-expressed.

Previous experimental studies have demonstrated that treatment with monoclonal antibodies directed against EGFR and small molecule inhibitors of the kinase have antitumor effects in various malignancies (58).

ERBB2 (Human Epidermal Growth Factor Receptor 2):

Increased expression of ERBB2 signaling has been demonstrated as a negative prognostic factor in KRAS wild-type mCRC patients treated with cetuximab or panitumumab.

EGFR sensitization to anti-EGFR agents has been found achievable with the help of dual-targeting agents aimed at both EGFR and ERBB2 (59).

There is evidence that treatment of breast and colorectal cancers exhibiting ERBB2 amplification with ERBB2 targeted drugs such as trastuzumab and pertuzumab when used in combination with EGFR inhibitors may improve outcomes (60).

Indeed, although PDGFRB mutations are not often mentioned, EGFR and ERBB2 are involved in cancer research and targeted treatment. These receptors are signaling coupled and may be targeted separately due to their cross-inhibition mechanism of action (61, 62).

Biochemical biomarkers: These come from the chemical processes that occur in bladder cancer cells. Markers of oxidative stress and enzyme activity are two examples (63, 64).

Cellular biomarkers: These are developed from bladder cancer cells’ cellular properties. Cell size, shape, and structure are a few examples (65).

Metabolic biomarkers: These come from the way bladder cancer cells metabolize their materials. Amino acid levels, glucose, and lactate are a few examples. A metabolomic biomarker is a specific molecule that comprises changes in the concentration of metabolites in biofluids or tissues that reliably portray a particular disease state. For example, increased levels of lipids and amino acids have been proven to be associated with biomarkers for bladder cancer in urine. Some of the examples include phosphocholine levels where bladder cancer patients had higher values than healthy controls (66). This metabolite is generated in the turnover of cell membranes and is more abundant in rapidly proliferating cancer cells. It is for this reason that the present study was aimed at establishing whether analysis of phosphocholine in urine may help clinicians in the early diagnosis of bladder cancer compared to cystoscopic examination or radiological imaging. Additionally, the variation in phosphocholine concentration in the course of the disease process may act as an indicator of disease activity or treatment efficacy. These metabolomic biomarkers shed hope of enhancing the levels of early diagnosis in bladder cancer patients and providing efficient treatment plans (67, 68).

Immunological biomarkers: These come from the immune system’s reaction to bladder cancer. Antibody reactions and cytokine levels are two examples (69).

Genetic biomarkers: These originate from genetic changes in bladder cancer cells’ DNA and RNA. Examples include chromosomal instability, expression levels of microRNA (miRNA), and mutations in the BRCA1 and BRCA2 genes (70).

Pan-cancer biomarker: The identification of biomarkers of multiple cancer types has been facilitated by the pan-cancer approach. More recent articles have aimed at evaluating the genomic consequences, the RNA transcription profiles, and clinical connections in a really comprehensive manner for cancer types (71, 72). For example, genetic pan-cancer studies have involved disulfide ptosis-relevant gene set regression, mitochondrial DNA repair gene, and cupro ptosis/copper metabolism genotypes. These large-scale works are designed to reveal similarities and dissimilarities of biomarker patterns for different cancers. In this type of approach, huge population-based genomics data and computational approaches can be used in identifying potential biomarkers that can extend across different cancers. Furthermore, cross-cancer studies on the associations between genes and drugs have been elucidated and there is a better understanding of immunity response characteristics (73, 74). There is also the consideration towards integrating various omics level information and strengthening the/proposing the use of machine learning-based approaches in order to derive more stable biomarkers across cancer types. These strategies establish a view to potentially enhance early detection and stratification of cancer diagnosis that distinguish this work from preceding action plans (71, 75).

Each of these groups provides distinct insights into the molecular pathways behind bladder cancer and may be able to influence treatment, prognosis, and diagnostic approaches. However, because bladder cancer is so diverse, finding and validating trustworthy biomarkers continues to be difficult.

In another classification, we have separated different biomarkers based on the location of the tumor, which are introduced in the next sections and also described generally in **Table 2**.

**Table 2:** List of bladder cancer biomarkers separated by location in blood, urine, tissue.

Location	Biomarker	Description
Blood	Cancer-associated antigen (CAAg)	a protein secreted by some cancer cell types, particularly bladder cancer cells. It is frequently used as a marker for the advancement of cancer and can be found in the blood (112).
Blood	Cancer Cell Exosomes	Small vesicles released by cancer cells that contain proteins and other molecules. They are being researched more and more as possible biomarkers for bladder cancer since they can convey information about the kind and stage of the disease (113, 114).
Urine	Nucleic Acid Testing	checking urine samples for the presence of cancer cell DNA or RNA. This approach has demonstrated potential in identifying bladder cancer in its early stages and distinguishing it from other forms of the disease (115, 116).
Urine	Protein Testing	Urine samples containing specific proteins may be able to detect bladder cancer. For instance, individuals with bladder cancer had higher levels of urine-based human complement factor H-related protein (hcfHrp) (117–119).
Urine	Whole Genome Sequencing	Using this method, every DNA molecule in a sample is examined to find any mutations that might point to bladder cancer. Urine samples have been used to diagnose urothelial carcinoma, a prevalent form of bladder cancer (120).
Tissue	Immunohistochemistry	This test looks for specific proteins in tissue samples using antibodies. Antibodies directed against specific proteins, for instance, can be used to identify bladder cancer cells in tissue samples (121, 122).
Tissue	Cytology	This entails using a microscope to examine a sample of cells to search for any anomalies that might point to bladder cancer (123, 124).
Tissue	Molecular Classification	This entails examining the genetic composition of bladder cancer cells to categorize them into several categories according to their molecular traits (125, 126).

##### 3.2.1.1 Tissue biomarker in bladder cancer

In tissues, there are Biomarkers such as Immunohistochemistry, Cytology, and Molecular Classification. Immunohistochemistry uses antibodies to detect specific proteins in tissue samples. Cells under a microscope can be used to detect abnormal cells which may signify bladder cancer during cytology. Molecular classification involves looking into the genetic composition of bladder cancer cells with a view of identifying them into different types based on their molecular features (76, 77).

##### 3.2.1.2 Body fluids biomarker in bladder cancer

Blood, urine, and tissue biomarkers aside; there are also others found in other body fluids. One such biomarker is Extracellular vesicles which are tiny particles released by cancer cells that can give information about the type and stage of cancer. They can be collected from different body fluids including Cerebrospinal Fluid (CSF), Tears, Amniotic Fluid, Peritoneal Fluid, Pleural Fluid, Aqueous Humor, and Synovial Fluid (78, 79).

Another example of biomarkers in other body fluids is plasma membrane protein analysis. This involves studying the proteins on the surface of cancer cells that can give clues about the type and stage of the cancer (80). Also, surfactant protein D analysis among others serves as a biomarker for other body fluids. Often, this protein is overproduced in cancer cells making it a possible sign of the presence of cancer (81). Also, cell-free DNA analysis is another biomarker found in other body fluids. It includes testing for extracellular DNA which may indicate the presence of cancer (82, 83). Additionally, viral load analysis can be considered as another biomarker that can be found in other body fluids (84). It includes an identification strategy to reveal the existence of viruses that sometimes cause or contribute to developing cancers. It is important to remember that these tests are not definitive although they produce useful information regarding cancer occurrence and advancement through their application by physicians diagnosing this ailment (85). They should also be used together with other diagnostic methods and clinical assessments since none of them alone can detect all cancers. Again, how effective these markers will be varies depending on bladder carcinomas histology subtype, disease stage, and general health status of a patient involved; thus necessitating personalization.

###### 3.2.1.2.1 Blood biomarker in bladder cancer

In blood samples, there exist some biomarkers like Cancer-associated antigen (CAAg) and Cancer Cell Exosomes, Carcinoembryonic Antigen (CEA), Prostate-Specific Antigen (PSA), Tumor Markers, Cytokines, Antibodies and MicroRNAs (miRNAs) (62, 86).

CAAg is a protein produced by certain types of cancer including those associated with bladder cancer. It is traceable within the blood and therefore used as an indicator for the development of cancer in many cases. Some information is available about the applications of cancer-associated antigens (CAAs) and exosomes in blood biomarkers for bladder cancer, however, these data need further confirmation. Tumor markers are any substances present on the membrane of cancer cells or any biomolecules released in the bloodstream. Their detection has mainly relied on different immunoassay methods; ELISA or FACS. For example, carcinoembryonic antigen is widely mentioned as an example of a general tumor marker and any selectivity for bladder cancer is low. Other bladder-specific markers have since come up and their test has been approved by the FDA; these include BTA stat and BTA TRAK. These tests include quantifying the levels of BTA in urine samples as an index of bladder tumor occurrence. However, this has some disadvantages because in inflammatory or other conditions are often highly positive (78).

In addition, exosomes-small extracellular vesicles secreted by cells, are also prospective blood biomarkers. They contain proteins, lipids, and nucleic acids that are derived from the parental cell and the profile matches that of the tumor cell. Methods of detection of exosomes in blood include size exclusion chromatography, ultracentrifugation, and flow cytometric analysis. Exosomal proteins (markers) involving CD63, TSG101, and ALIX are commonly widely used for identification of the exosome. Such exosomes may have distinct profiles of proteins; the profiling of proteins associated with such exosomes may be characteristic of bladder cancer (87). For example, EGFR, HER2, or CA125 can be selected protein markers of bladder cancer on exosomes. Standardization and improvement of the sensitivity and specificity of liquid biopsy approaches using exosomes for the noninvasive diagnosis of bladder cancer is still a challenge. New findings in nanotechnology and flow cytometrics have made it possible to gain a better understanding of exosomes by paying basic methods to disease diagnostics. However, the identification of pure populations of tumor-derived exosomes from heterogeneous populations in blood is considered a major challenge (88).

Cancer Cell Exosomes are minute vesicles released from cancer cells containing proteins among other molecules. They are capable of carrying details that reveal what kind and how far along it has gone and thus they have been increasingly studied as possible markers for bladder cancers (89).

Carcinoembryonic Antigen (CEA) is a protein manufactured by malignancy cells, especially those in the colon and anus. It is among the most generally used biomarkers to detect colorectal cancer at its early stages (90).

###### 3.2.1.2.2 Urine biomarker in bladder cancer

Urine is subjected to the usage of biomarkers like Nucleic Acid Testing, Protein Testing and, Whole Genome Sequencing. The testing involves checking urine samples for DNA or RNA from cancer cells. Certain proteins in urine can indicate blabber cancer; all these proteins are detected during protein testing. Whole genome sequencing entails analyzing all the DNA present in a sample to identify mutations indicative of bladder cancer. The nuclear matrix protein BLCA-4 (BLCA-4 NMP) is one such biomarker (91, 92). Research has indicated that patients with bladder cancer may have considerably different urine levels of BLCA-4 NMP than patients without the disease. As a result, variations in BLCA-4 NMP levels may act as a biomarker for bladder cancer. The protein hyaluronic acid, which is present in numerous bodily fluids, is another possible biomarker (93). Urine hyaluronic acid levels are greater than normal in cases of bladder cancer. Thus, a higher-than-normal concentration of hyaluronic acid in the urine may be a biomarker for bladder cancer. Additionally, exosomes—tiny vesicles secreted by cancer cells—have been investigated as possible bladder cancer indicators. Although the extraction of exosomes from urine is still in its infancy, early research indicates that exosomes may include distinct markers that indicate bladder cancer. It’s crucial to remember that even though these putative biomarkers appear promising, research on them is still ongoing, and they are not yet generally acknowledged as trustworthy instruments for bladder cancer diagnosis. To validate these biomarkers and comprehend their possible benefits and drawbacks, more research is required (94, 95).

Urine biomarkers have been widely studied for bladder cancer diagnosis because they are minimally invasive and can be used in the early stage. However, these soluble biomarkers must be compared with their equivalent in the blood for their sensitivity, specificity, and clinical usefulness. In examining the efficacy of urine-based molecular diagnostics compared to blood-based molecular diagnostics for bladder carcinoma there are several studies. For example, a meta-analysis that appeared in the Journal of Urology in 2019 sought to compare the diagnostic accuracy of markers in urine (NMP22, BTA stat, and BTA TRAK) against blood-based markers (CEA and CYFRA21-1) (96). The findings revealed that there was desirable performance, but blood and urine biomarkers exhibited moderate sensitivity and specificity, compared to each other; nonetheless, urine tests were slightly superior to blood-based diagnosis, especially in differentiating NMIBC. Research conducted in clinical chemistry in 2023 showed that NMP22, BTA stat, and uPA urine biomarkers were better than CEA and CYFRA 21-1 blood markers in the differentiation of bladder cancer patients and controls, especially among high-risk populations (97).

However, seeing that in other sorts of cancer including neuroblastoma liquid biopsy is used more and more in diagnosing cancer, it is still relatively sparingly utilized in cases of bladder cancer. Regarding bladder cancer, urine-based biomarkers continue to be the most researched approach to mimicking liquid biopsy-like diagnostics. The advantages include easier to collect, cheaper than blood test, and to determine specific molecular alteration in bladder epithelial cells. However, some remain and they are The following; There is still some question of how to standardize the SOPs for urine collection and adequacy performance across multiple labs. Such problems are still being studied at present to diagnose bladder cancer through liquid biopsy and in the near future they may bring more advanced forms of liquid biopsy to better early detection and prognosis of the cancer (98).

### 3.3 Common biomarkers in blood, tissue, and urine in bladder cancer

MicroRNAs (miRNAs) are essential for many biological processes, including the initiation and spread of cancer. It has been shown that several miRNAs may serve as bladder cancer biomarkers (99). Here are a few of them:

miR-20a: According to reports, the miRNA in question is overexpressed in tissues from bladder cancer, suggesting that it could be used as a diagnostic marker (100).

miR-155: Its upregulation in bladder cancer cells suggests a role for it in the disease’s progression (101).

miR-21: According to reports, the expression of this miRNA is markedly elevated in bladder cancer tissues, suggesting that it may have predictive value (102).

miR-133b: Due to its high expression in bladder cancer tissues, this miRNA may have a role in the development of tumors (103).

miR-15b: According to reports, the expression of this miRNA is markedly elevated in bladder cancer tissues, suggesting that it may have predictive value (104).

UCA1: It has been proposed that this lncRNA can potentially be used as a marker for bladder cancer. It interacts with several proteins and pathways involved in the progression of bladder cancer and is upregulated in bladder cancer tissues and cell lines (105).

H19: Another possibility of this lncRNA being a bladder cancer marker is looked at. It has been seen to interact with p53 and miR-29b-3p, two crucial factors in bladder malignancy development (106).

TUG1: This lncRNA has been associated with invasion, migration, and proliferation of the bladder due to inhibition of miR-29c (107, 108).

CALML3-AS1: Bladder cancer is linked to this lncRNA’s interaction with ZBTB2 and miR-4316 (109).

NEAT1: Bladder Cancer has been linked to this lncRNA through its interaction with HMGB1 and miR-410 (110).

lnc-MUC20-9: It was observed that this particular lncRNA is overexpressed in urinary bladder carcinoma; however, its exact function and importance remain unknown. These LncRNAs are being vigorously investigated for their potential use as biomarkers of bladder cancer. Such a discouraging result is still encouraging, more investigations should be done to validate them as diagnostics and also comprehend their role in the onset of bladder cancer (111).

### 3.4 Biomarkers in bladder cancer diagnosis

There are now several promising markers in the field of bladder cancer biomarkers. For example, it has been demonstrated that urinary BTA signals bladder malignancy. Similar to this, research has been done on ImmunoCyt, a novel monoclonal antibody test for bladder cancer (127).

The identification of a novel mucin antigen, MAUB, linked to bladder cancer, is another noteworthy discovery (128). These indicators offer promising new opportunities for bladder cancer diagnosis and early detection But there are still difficulties despite these encouraging advancements. The necessity for additional validation of these biomarkers in bigger patient cohorts to verify their usefulness and dependability is one of the main challenges. Critical factors to take into account are also those about cost, accessibility, and the possibility of false positives or negatives.

Prospective investigations may concentrate on incorporating these biomarkers into current diagnostic instruments, creating novel diagnostic algorithms, and carrying out extensive clinical trials. Additionally, studies may look into the possibility of integrating biomarkers with conventional diagnostic techniques to provide a more thorough and precise diagnosis. Additionally being investigated is the possibility of using exosomes and extracellular vesicles (EVs) as biomarkers for bladder cancer (129, 130). These little particles, secreted by cancerous cells, include an enormous variety of proteins that may act as distinct indicators for this illness.

To establish standardized procedures and prove their diagnostic value, more research is necessary in the still-developing field of urine particle isolation. Long non-coding RNAs (lncRNAs) may perhaps play a more significant role in bladder cancer in the future. LncRNAs are a class of RNAs that are essential for the regulation of genes but do not code for proteins. According to reports, several lncRNAs are increased in bladder cancer cell lines and tissues, which raises the possibility that they could be biomarkers (131, 132). Nevertheless, research on lncRNAs as biomarkers is currently ongoing, and more investigation is required to validate their diagnostic utility and comprehend their functions in the development of bladder cancer. Another class of RNAs that has been suggested as a possible biomarker for bladder cancer is called microRNAs (miRNAs). Small non-coding RNAs known as miRNAs control post-transcriptional changes in gene expression (133). It has been revealed that bladder cancer tissues overexpress several miRNAs, suggesting that they could serve as useful diagnostic markers. Nevertheless, research on miRNAs as biomarkers is currently ongoing, and more investigation is required to validate their diagnostic utility and comprehend their functions in the development of bladder cancer (134).

Beyond this, research is also being done on the possibility of using proteins as biomarkers for bladder cancer. Large molecules called proteins carry out a variety of tasks in the body. It has been observed that several proteins are overexpressed in bladder cancer tissues. These proteins may function as disease biomarkers, but more investigation is required to confirm their diagnostic value and comprehend their functions in the development of bladder cancer. It’s crucial to remember that these biomarkers have limits despite their potential. For example, these biomarkers may not be appropriate for all individuals and their sensitivity and specificity may change based on the stage and subtype of bladder cancer (135).

As such, these considerations must be taken into account when choosing and evaluating biomarkers for bladder cancer. Although bladder cancer biomarkers have demonstrated significant potential, further investigation is required to harmonize the testing protocols and validate these biomarkers in a clinical context. This will open the door to bladder cancer diagnosis and surveillance that is more precise and effective, ultimately leading to better patient outcomes. Subsequent investigations may concentrate on incorporating these biomarkers into currently available diagnostic instruments, creating novel diagnostic algorithms, and carrying out extensive clinical trials. Additionally, studies may look into the possibility of integrating biomarkers with conventional diagnostic techniques to provide a more thorough and precise diagnosis (136, 137).

### 3.5 Biomarkers in bladder cancer prognosis and prediction

Some tumor tissue biomarkers can tell how aggressive a certain cancer is or reveal if the disease is expected to recur. EGFR gene expression, for instance, which tends to be overexpressed in bladder cancer, is one such biomarker (138). However, an equally high level of EGFR expression can signal poor prognosis since it is linked to more severe diseases. Again, PDGFRA gene expression which usually mutates in bladder cancer may foretell a worse prognosis as well (139, 140). Thus this way doctors can plan treatment options taking into account the tumor’s characteristics thereby possibly improving patients’ lives.

Another emerging prognostic biomarker for bladder cancer is tumor-infiltrating lymphocyte (TIL) count (141). A higher TIL count means that a tumor has become more aggressive thus indicating less favorable outcomes (142). Immune response against cancers involves TILs and they are found within the microenvironment of tumors that impact on their behavior and reaction toward treatment methods (143, 144).

### 3.6 Bladder cancer kits and test

UBC^®^ Rapid tests and UBC ELISA test kits are urine biomarkers that help to detect bladder cancer early. Rapid UBC^®^ is a point-of-care (POC) testing for the fragments of cytokeratins 8 and 18 in urine which are overproduced in bladder cells suffering from this disease. The UBC ELISA kit is an enzyme-linked immunosorbent assay that measures the level of these fragments in the urine (145). These tests have shown good sensitivity and specificity in detecting bladder cancer, particularly in patients with non-muscle-invasive high-grade tumors. They do however have some drawbacks such as false positives and the need for sophisticated equipment and trained personnel (146, 147). The UBC^®^ Rapid test and the UBC ELISA are immunological tests for the detection of bladder carcinoma which are performed directly on urine samples. Thus, their performance is considered to be closely related to cystoscopy, which is widely used as the gold standard for the diagnosis of bladder cancer. Their precision, reactivity, and selectivity are essential to determine to consider their utility in clinical practice (148).

Sensitivity: Of the existing literature, the sensitivity of the UBC^®^ Rapid test has been observed to vary. For example, its ability to diagnose CIS patients with 86.9 and NMIBC with 71.4% of high grade without muscle invasion. However, a meta-analysis reported a pooled sensitivity of 59% (95% CI: The overall range was between 55 – 62% through the various studies.

Specificity: It seems that the specificity of UBC^®^ Rapid assay is usually high and has been estimated to be 90.9% by several researchers. In contrast, the meta-analysis indicated a pooled specificity of 76% (95% CI: 72–80%).

Comparison to Cystoscopy: Even though cystoscopy is the gold standard in diagnosing these lesions, sensitivity was improved to 95.8% using UBC^®^ Rapid with other diagnostic modalities like ultrasound and cytology though the specificity dropped to 67.3%.

While performance metrics such as elasticity, recovery rate, and cut-off factors were not featured in the marketed search results, UBC ELISA is observed to have comparable diagnostic outcomes to those of the UBC^®^ Rapid assay but may differ with certain clinical situations and patient groups (149).

As we showed, the sensitivity and specificity of both tests can be variable, meaning that although they may be useful for first-line screening, they should not replace cystoscopy. It may therefore in fact be better considered in the context of being complementary tests that may be useful in risk staging or to guide further assessments (150).

1-Non-invasive Detection Study: The accuracy of UBC^®^ Rapid in several bladder cancer conditions was established in this study with emphasis on the sensitivity and specificity of the technique as compared to cystoscopy and other techniques.

2-Meta-analysis: Regarding diagnostic accuracy, the efficiency of the UBC^®^ Rapid test obtained using multiple studies was calculated for sensitivity and specificity, and more research using larger samples was recommended (151).

3-Comparative Study: Urothelial brushing and washing study against urine cytology indicates that UBC^®^ Rapid increases sensitivity and decreased specificity thought to have potential in tandem with other technologies.

Thus, although the performance of both UBC^®^ Rapid and UBC ELISA is useful in the assessment of bladder cancer, assessment of its effectiveness has revealed that it is better utilized in conjunction with conventional diagnostic procedures such as cystoscopy to give an accurate description of the patient (146).

Xpert^®^ Bladder Cancer Monitor is a portable gadget, on examines a drop of urine will determine if there is any presence of bladder cancer. Using spectrophotometric, electrochemical, and colorimetric technologies combined, this device measures specific bio-markers concentration in the urine (152). This product has a user-friendly nature; hence anyone can be able to use it without going through intense training making it suitable for healthcare providers working in remote areas or those who lack resources. However, like other urinary biomarkers, it has limitations regarding its sensitivity/specificity and cannot be used instead of cystoscopy to diagnose definitively bladder cancer (153). The BC UroMark is a panel of urinary biomarkers that comprise tests for bladder cancer, bladder stones, and interstitial cystitis. In this way, it combines the best features of several tests into one panel to give a thorough assessment of the health of your bladder (154). However, some limitations include the potential for false positive and false negative results as well as the need for specialized equipment and trained personnel.

One kind of nucleic acid-based test that may identify and measure the presence of particular DNA sequences in a sample is TaqMan^®^ Arrays. TaqMan arrays can be used to find mutations in genes, like the EGFR and KRAS genes, that are commonly changed in bladder cancer cases (155). This can help inform therapy options and provide important information about the genetic makeup of the malignancy. Like other genetic tests, it has drawbacks, though, such as the possibility of false positives and negatives and the need for specialized tools and workers with the necessary training (156). Both EGFR and PDGFRA act as managing obvious prognostic factors in many cancers including glioblastoma. For instance, it has been found that changes in these genes are associated with tumor localization and the patient’s outcome. For instance, EGFR alterations are related to cortical lesion originating and PDGFRA alterations with multiple lesions, data helpful for treatment selection and prognosis. EGFR and PDGFRA testing results are used in the clinic for guiding decisions on surgical resection and targeted therapy application. For instance, patients with co-amplification of these receptors should be considered for agents that block their signal transduction (157). Additionally, Ki-67 labeling index is higher in tumors with PDGFRA alterations, which could have impacts on adjuvant therapy decisions. Currently, there is increasing evidence about the association between EGFR overexpression and the response to treatment in bladder cancer. Some prior research has looked at the relationship between it and EGFR expression; the results propose that a high sign for EGFR is related to low popularity and resistance to some treatments. For example, one research suggested that patients with bladder cancer, who transmitted EGFR overexpression, had poor response toward chemotherapy, indicating the importance of new treatments (58, 158).

Also, there are more current studies demonstrating EGFR inhibitors in the treatment of bladder cancer in combination treatment with conventional treatments. The compiled effects of these findings give an indication that overall EGFR detection may be vital in prescribing personalized treatment schedules. Not only EGFR and PDGFRA, but other novel biomarkers are receiving recognition for bladder cancer. Among the abovementioned targets, important findings include recent research simply known as “Data-mining-based biomarker evaluation and experimental validation of SHTN1 for bladder cancer” published in 2024 (159).

Accordingly, this study suggests that SHTN1 may be involved in the development of cancer in the context of tumor growth and may be an imperative marker for treatment.

To that end, the discovery and subsequent confirmation of these biomarkers are critical for enhancing multifactorial treatments of bladder cancer within the paradigm of personalized medicine, as they give more precise information on how to deal with an individual tumor.

A soluble version of the protein fibronectin, which is involved in cell adhesion and cellular dissemination, is called soluble FAS (sFAS). Elevated sFAS levels have been reported in bladder cancer, suggesting that it could be a biomarker for the illness (160). It does have certain drawbacks, though, such as the possibility of false positives and negatives as well as the need for specialized tools and qualified staff. One type of fibronectin that is overproduced in bladder cancer is called bladder tumor fibronectin (BTF) (161, 162). It has been suggested as a possible biomarker for this illness, but there are drawbacks, such as the possibility of false positives and negatives and the need for specialized tools and qualified staff.

Two proteins that are overexpressed in bladder cancer are IGF2 and MAGE-A3 can detected by(Immunohistochemistry (IHC) or Western Blotting or Quantitative PCR (qPCR)/RT-PCR) (163, 164). Because of their limitations, these indicators need more investigation and validation, even if they have demonstrated promise in the diagnosis and prognosis of bladder cancer. To increase the precision and dependability of the diagnosis, they ought to be utilized in concert with additional diagnostic techniques like cystoscopy (42). For each method, specific data on sensitivity and specificity would be required to make a comparison table between cytology and commercially available biomarker tests for bladder cancer.

Specificity is the percentage of actual negative cases that are correctly identified, whereas sensitivity is the percentage of genuine positive cases that are identified correctly. Variations in research sizes, assay cut-points, and the grade and stage of the malignancies under examination are reflected in the range of percentages (165, 166).

Two primary measures that are used to assess the effectiveness of diagnostic tests are sensitivity and specificity which in **Table 3** shows the approximate value of these two components for known tests. Sensitivity, also referred to as true positive rate (TPR), quantifies the percentage of real positive cases (those who have the disease) that are correctly identified by the test. For instance, if a test has a sensitivity of 87%, it indicates that 87 out of every 100 individuals with the disease will be correctly identified by the test. Conversely, specificity, which is often referred to as true negative rate (TNR), quantifies the percentage of real negative cases—that is, individuals who do not have the disease—that the test accurately identifies (165, 167). If a test, for example, has a 96% specificity, it means that, out of 100 persons, 96 will not be misidentified by the test as not having the condition. Varying diagnostic techniques can have quite varying sensitivity and specificity ranges. As an illustration, urine microscopy, a widely used technique for identifying bladder cancer, has a 92–96% specificity range and an 87-91% sensitivity range. This implies that even if this approach is quite good at identifying people who have the illness, it might not always identify people who don’t. Another technique for detecting bladder cancer is urine cytology, which has a greater specificity range of 73–100% and a lower sensitivity range of 13.3–86%. This shows that it is quite good at correctly identifying those who do not have the disease, even though it may not be as good at identifying those who do (165, 168).

**Table 3:** The diagnostic method ranges for urine microscopy, urine cytology, urine markers, cystoscopy, CT scans, MRIs, and a combined urine markers and urine cytology method are displayed in this table.

Test	Sensitivity Range	Specificity Range
Urine Microscopy	87-91%	92-96%
Urine Cytology	13.3-86%	73-100%
Urine Markers	45.4-100%	12.1-97.2%
Cystoscopy	68.3-100%	57-97%
CT Scan	46-86.7%	77.8-100%
MRI	78.2-87.5%	77.8-93.3%
Urine Markers + Urine Cytology	61.9-94%	50-90%

There is a wide variety of sensitivity and specificity values for urine markers, which are compounds that may be detected in the urine and can indicate the presence of specific diseases. Depending on the population under study and the particular marker being evaluated, these can differ significantly (169). Certain markers, for instance, may be highly sensitive but poorly specific, which means they may accurately identify people who do not have the disease while simultaneously being effective at detecting those who do. Some people may be skilled at correctly identifying those who do not have the condition but may overlook those who do. This is known as high specificity but low sensitivity (168, 170).

## 4 FDA cancer treatment

In the past few years, the United States Food and Drug Administration has instituted many promising treatment modalities for bladder cancer, which has broadened the options available for patient management. Such approvals are indicative of improvement in the existing knowledge about the disease as well as the discovery of newer treatment models (171).

Key FDA-approved treatments for bladder cancer include:

1. Immunotherapy combinations:

N-803 (Anktiva) with BCG in BCG-unresponsive NMIBC.

Enfortumab vedotin (Padcev™) in combination with pembrolizumab (Keytruda ^®^) for newly diagnosed patients with metastatic urothelial carcinoma 2 for a year 4 (172).

2. Chemotherapy combinations:

Gemcitabine cisplatin and MVAC (methotrexate, vinblastine, doxorubicin, cisplatin) (173).

These treatments therefore go a long way in improving bladder cancer management. For instance, the approval of N-803 in conjunction with BCG has created a therapeutic option for patients with BCG non-responsive disease, many of whom may avoid invasive procedures including cystectomy. Likewise, enfortumab vedotin and pembrolizumab are also a feasible first-line therapy for metastatic bladder cancer that are not adequate for chemotherapy (174).

The recent approvals by the FDA show how quickly the world of bladder cancer treatment is changing. They map the evolution of immunotherapy in the context of bladder cancer and show that researchers are continuing to work on strategies that will help enhance the efficacy of existing treatments for all stages of the illness (175).

## 5 Novel treatment approaches

Large-scale clinical trials, the creation of reliable diagnostic algorithms, and the incorporation of these biomarkers into currently available diagnostic instruments will also be necessary. By doing this, we may maximize the potential of biomarkers for bladder cancer and greatly enhance patient treatment. An important tactic in the effort to enhance bladder cancer detection and treatment is the incorporation of these biomarkers into the current diagnostic apparatus. This might entail creating new algorithms that combine the advantages of several biomarkers to boost the test’s sensitivity and specificity (176, 177).

A more complete image of the illness may be provided by such integrated systems, enabling more precise diagnosis and individualized treatment regimens. Moreover, investigating the possibility of integrating biomarkers with conventional diagnostic techniques may result in a diagnosis that is more precise and thorough. For example, the combination of biomarker testing with imaging technologies may improve the accuracy of the diagnosis, especially when the biomarkers do not yield a conclusive result (64). In a similar vein, the combination of genomic profiling and biomarker testing may offer insightful information on the genetic makeup of bladder cancer, which may help determine the best course of treatment and forecast patient outcomes. There are many obstacles to fully realizing the promise of bladder cancer biomarkers, but the rewards might be enormous. These biomarkers have the potential to completely change how bladder cancer is identified and treated, resulting in earlier diagnosis, better treatment results, and eventually higher patient survival rates. More study and innovation in this area is needed. Thus, we must tackle the related difficulties and carry out more research and validation of these biomarkers (65).

Other study topics that are just as crucial include creating novel treatment approaches, bettering patient care, and comprehending the molecular causes of bladder cancer. Comprehending the molecular pathways behind bladder cancer, for example, can help explain why certain individuals react well to treatment while others do not. This information can direct the creation of more potent and minimally side-effect-tailored medicines. In a similar vein, boosting patient-provider communication, offering emotional support, and making sure patients have access to the newest treatment options can all contribute to better patient care (64). Furthermore, biomarker validation is a collaborative effort. It has to be a component of a larger initiative to enhance bladder cancer detection, staging, and care. This includes creating registries to track the effectiveness of these biomarkers over time, implementing standardized guidelines for the use of biomarkers, and developing novel diagnostic tools. In summary, although the identification and verification of biomarkers for bladder cancer constitute a noteworthy advancement, they remain but a single component of the puzzle. To effectively revolutionize bladder cancer management, a holistic strategy that encompasses studying the disease’s molecular processes, creating new therapeutic approaches, and enhancing patient care is required. The identification and validation of biomarkers represent a major advancement in the rapidly changing field of bladder cancer research. However, it’s crucial to keep in mind that these biomarkers are only a single component of the picture. While they can be useful in the early identification and diagnosis of bladder cancer, they cannot take the place of a thorough medical examination (178).

Clinical judgment should always direct the use of these biomarkers, and their interpretation should always be considered in conjunction with other diagnostic data. For example, a patient’s positive biomarker test result does not always indicate bladder cancer. Either a false positive or an indication of another illness that needs to be ruled out could be the cause. As a result, before beginning therapy after a positive biomarker test, a patient should speak with a healthcare professional who can weigh all the information at hand. Furthermore, these indicators have limitations even if they have the potential to enhance bladder cancer detection and diagnosis. These include the risk of false positives or negatives, the necessity for additional validation in larger patient cohorts, and cost and accessibility concerns (179). It will take coordinated efforts from researchers, physicians, and politicians to address these issues. Although the identification and verification of biomarkers for bladder cancer constitute a noteworthy advancement, they remain but a single component of the puzzle. To effectively revolutionize bladder cancer management, a holistic strategy that encompasses studying the disease’s molecular processes, creating new therapeutic approaches, and enhancing patient care is required (178).

We point out how machine learning methods may be able to increase these biomarkers’ prediction accuracy. Biomarker testing may become more sensitive and specific thanks to machine learning’s capacity to learn from and make predictions based on data. But this also calls into question how interpretable and explainable these models are, which are crucial issues to take into account considering how they can affect patient treatment (180). To expedite the translation of these biomarkers into clinical practice, we also support cooperative efforts among researchers, doctors, and patients. In order to turn scientific findings into useful applications that can help patients, collaboration is essential. It entails pooling resources and expertise, organizing activities, and cooperating to overcome obstacles and accomplish common objectives (181, 182).

In general, different approaches are used to diagnose bladder cancer as well as to predict and follow treatment. **Table 4** shows Current Approaches and Future Directions in the diagnosis, prognosis, and treatment of bladder cancer.

**Table 4:** Display current Approaches and future research in diagnosis, prognosis, and treatment of bladder cancer.

Current Approaches	Future Directions
FDA-approved molecular biomarkers including BTA stat, BTA TRAK, and NMP22	Archiving the biomarkers into current diagnostic tools
ImmunoCyt is a further monoclonal antibody test that is used to monitor bladder cancer.	Inventing/producing new diagnostic algorithms
MAUB^2^ is a newly found mucin antigen that is linked to bladder cancer	Doing large clinical trials
Extracellular Vesicles (EVs) and Exosomes	Exploiting the possibilities of combining biomarkers with traditional diagnosis methods
Studies the Role of Long Non-Coding RNAs(lncRNAs) in Bladder Cancer	Creating a standardization for testing procedures and validating these markers in clinic settings
overexpression of specific miRNAs in bladder cancer tissues	Mentioning issues on cost, access as well as false negatives or positives among other problems.

## 6 Challenges in the biomarker field

Molecular heterogeneity: Bladder cancer has the highest degree of molecular and clinicopatologic diversity which makes it difficult to formulate universal biomarkers.

Limited predictive biomarkers: Some prognostic biomarkers are available; however, predictive biomarkers that assist in effective therapy selection are urgently required.

Discordance between classification schemes: Different molecular categorization systems of bladder cancer hampers their feasible application.

Characterizing the DNA damage response (DDR) axis: Gene rearrangements and their mutations in the DDR genes show mixed evidence about their use as biomarkers of chemotherapy sensitivity (183).

Biomarkers for emerging targets: With emerging new targets (for example, Nectin-4), there is an equally important need to develop relevant biomarkers.

Funding constraints: The availability of patients for clinical studies and limited funds constrain the advancement and clinical utility of biomarkers.

Variability in trial endpoints: Variations in the endpoints employed in many clinical trials hamper the comparability of results across trials.

Standardization needs: Assay standardization is necessary to ensure that consistency is achieved across studies.

Data integration challenges: The widespread adoption of multi-omics has made it increasingly difficult to incorporate data from a range of sources.

Regulatory hurdles: getting to the level where there is a high possibility that biomarkers can be used in clinical settings as now their clinical development has various complexities (184–186).

## 7 Clinical trials on the biomarker diagnosis

Bladder cancer diagnosis has been linked to numerous studies examining various urinary biomarkers. One of the trials was based on the ADXBLADDER test, which aimed at detecting the mini-chromosome maintenance protein 5 (MCM5) in the urine. This test was reported to have a high sensitivity (76-95%) for high-risk and muscle-invasive bladder cancers and thus can replace or augment urine cytology. UroSEEK, which targets changes in multiple genes is another assay that yields interesting results. Clinical studies indicated better sensitivity than cytology for both surveillance (71% vs 25%) and diagnostics (95% vs 43%). CxBladder, an assay based on measuring specific mRNAs, also had high sensitivity (82-93%) and negative predictive values (96-99%) during clinical validation. Although these biomarkers provide a trouble-free means of examination as cystoscopies are avoided, their specificity is often lower than that of the more conventional techniques. Strategies to enhance sensitivity and decrease false positives for effective and practical clinical usage are in progress (79, 184).

## 8 Conclusion

Our review study concludes by highlighting the noteworthy advancements in the identification and validation of bladder cancer biomarkers. There are now more options for the early identification and diagnosis of this illness thanks to the development of newer biomarkers such as urinary BTA, the ImmunoCyt monoclonal antibody test, and the MAUB mucin antigen. However, there are many obstacles in the way of progress, most notably the requirement for more biomarker validation in bigger patient cohorts and the need to address concerns about cost, accessibility, and the possibility of false positives or negatives.

These biomarkers have enormous potential benefits despite these obstacles. They might completely alter the way bladder cancer is found and treated, which would result in earlier detection, better treatment results, and eventually higher patient survival rates. Ongoing research must address the related difficulties while simultaneously exploring and validating these biomarkers. Subsequent investigations ought to concentrate on incorporating these biomarkers into currently available diagnostic instruments, creating novel diagnostic algorithms, and carrying out extensive clinical trials. For a more precise and thorough diagnosis, studies may potentially examine the possibility of integrating biomarkers with conventional diagnostic techniques. By doing this, we may maximize the potential of biomarkers for bladder cancer and greatly enhance patient treatment.

Within the quickly developing field of bladder cancer research, the discovery of novel biomarkers is a noteworthy achievement. Promising pathways for early detection, diagnosis, and prognosis of bladder cancer are provided by these biomarkers, which include the UBC^®^ Rapid test, UBC ELISA kit, Xpert^®^ Bladder Cancer Monitor, BC UroMark, TaqMan^®^ Arrays, Soluble FAS (sFAS), Bladder tumor fibronectin (BTF), and IGF2 and MAGE-A3. They have the enormous potential to completely change the way bladder cancer is treated, which would result in earlier detection, better treatment results, and eventually higher patient survival rates. However, there is still a long way to go before these biomarkers can be fully utilized. We must face the issues that lie ahead as we proceed. These include potential false positives or negatives, cost and accessibility concerns, and the need for additional validation of these biomarkers in larger patient populations. It will take coordinated efforts from researchers, physicians, and politicians to address these issues.

We talk about the need for continued study in this area. Continuous research is crucial because cancer biomarker research is dynamic, with new biomarkers being found and existing ones being improved and validated. It will ensure that patients have access to the most precise and efficient monitoring and diagnostic tools possible by keeping up with changes in the field and technology. To sum up, the creation and verification of biomarkers for bladder cancer constitute a noteworthy advancement in the battle against this illness. They are only a single component of the puzzle, though. To effectively revolutionize bladder cancer management, a holistic strategy that encompasses studying the disease’s molecular processes, creating new therapeutic approaches, and enhancing patient care is required.

The creation and validation of biomarkers is only one piece of the jigsaw in the larger scheme of bladder cancer research. Other study topics that are just as crucial include creating novel treatment approaches, bettering patient care, and comprehending the molecular causes of bladder cancer. Comprehending the molecular pathways behind bladder cancer, for example, can help explain why certain individuals react well to treatment while others do not. This information can direct the creation of more potent and minimally side-effect-tailored medicines. In a similar vein, boosting patient-provider communication, offering emotional support, and making sure patients have access to the newest treatment options can all contribute to better patient care. Furthermore, biomarker validation is a collaborative effort. It has to be a component of a larger initiative to enhance bladder cancer detection, staging, and care. This includes creating registries to track the effectiveness of these biomarkers over time, implementing standardized guidelines for the use of biomarkers, and developing novel diagnostic tools.

1. Integration of AI and Machine Learning:

-Predictive Modeling: Future research should investigate how AI and ML algorithms can be used to process huge amounts of datasets from genomic, proteomic, and clinical bases to detect biomarkers known to trigger bladder cancer and predict prognosis to treatment. This could give rise to patient outcome prediction models which in turn inform the patient management strategy.
-Image Analysis: AI could enhance the analysis of imaging studies and histopathological slides to a large extent. For instance, machine learning can be effectively used to segment tissue images and detect changes in tissue architecture that point to malignancy, so that initial diagnosis of bladder cancer can be more accurate than with traditional methods.

2. Biomarker Discovery through Genomic Technologies:

-Next-Generation Sequencing (NGS): The application of NGS technologies could help in the identification of new biomarkers by offering a great potential handy understanding of genetic changes related to bladder cancer. It may reveal novel targets for diagnosis and therapy, which were not identified before using other strategies.
-Single-Cell RNA Sequencing: With this technology, it is possible to study cellular heterogeneity in tumors, and therefore theoretically identify markers of tumor microenvironment interactions and the formation of treatment resistance.

3. Validation of Emerging Biomarkers:

-Clinical Trials: Future clinical trials should be prospective studies to confirm the accuracy and precision of such novel biomarkers as UBC^®^ Rapid, URO17, or other biomarkers described in this manuscript. Performing these trials should preferably be so in a variety of population types and clinical environments to increase external validity.
-Comparative Effectiveness Research: Subsequent studies should address the steps of evaluating the new biomarker tests about the conventional diagnostic measures such as cystoscopy and urine cytology to understand their place in the clinical practice.

4. Utilization of Multi-Omics Approaches:

The integrated analysis of the genomics, proteomics, and metabolomics of bladder tumors will give a comprehensive assessment of the function of this malignancy. Further clinical investigations should be focused on analyzing these multi-omics datasets together to find better biomarker signatures for better diagnostic performance and risk classification.

5. Patient-Centric Approaches:

Exploring patient-reported outcomes in collaboration with biomarker testing may help add to what is known about the effects of these interventions on quality of life and treatment decisions. Future work should aim towards identifying molecular markers for cancer early diagnosis and prognosis as well as the patient’s response to particular treatments.

Including these future research directions in the conclusion, part will give a better picture of how the improvement in technology can improve biomarker-based diagnostics for bladder cancer. Whereas focusing on the updates and conceiving your article as a theatre of occasional citations can limit the scalability and timeliness of your review, emphasizing AI, machine learning or multi-omics approaches will keep your review relevant to the current active debates in the rapidly progressing field. Although the necessity of more substantial validation of bladder cancer biomarkers is recognized, practical recommendations can strengthen the call for the use of biomarkers in clinical practice. Here are specific approaches that could be employed to strengthen the validation process:

1. Multi-Center Trials

It is for this reason that multi-center trials have to be carried out to determine inter-center variability and reproducibility of different biomarkers. Such trials can:

Enhance Sample Diversity: To capture the extensive variability of users’ behavior, more diverse participants with different demographics and clinical histories will be included in the study.

Standardize Protocols: That is, through applying the same approach to various centers, it is possible to reduce the variation in the data to an extent, which will enable researchers to draw more accurate conclusions as to the effectiveness of a biomarker.

2. Compatibility with the Current Diagnostic Modalities

The application of biomarker tests, used together with more conventional diagnostic techniques such as cystoscopy will provide more precise results. Specific strategies include:

Sequential Testing: Adoption of a scanning profile in which biomarker tests are performed with cystoscopy only on those who test positive for the disease will help to prevent the overuse of invasive examinations.

Complementary Use: Combined with biomarkers, cystoscopy can identify patients with an increased risk of malignancy, which will help to tailor the surveillance and therapy. For instance, researchers have presented the use of UBC^®^ Rapid in conjunction with cystoscopy to increase the sensitivity of the latter up to 95.8% in identifying bladder cancer without the cystoscopy procedures alone. 23

3. Comparative Effectiveness Research

In conducting comparative efficacy studies it will be possible to compare the new biomarkers to other diagnostic approaches currently in use. This could involve:

Head-to-Head Studies: A direct comparison of new biomarkers such as UBC^®^ Rapid and NMP22 with urine cytology and cystoscopy based on their sensitivity, specificity, and diagnostic efficacy.

Longitudinal Studies: Using patients followed over time to determine, how accurately these biomarkers predict recurrence or progression compared with current reference standards.

4. Use of Advanced Technologies

Incorporating cutting-edge technologies such as AI and machine learning can enhance biomarker validation:

Data Mining and Pattern Recognition: The possibilities of AI algorithms are in analyzing the data from multi-center trials to find out something that can be missed when using the conventional statistical approaches.

Predictive Modeling: Algorithms can then be built to use biomarkers in conjunction with other clinical data to not only help identify important determinants for patient prognosis but also to refine treatment recommendations.

5. North America remains the most dominant region while the least active region is Asia.

To facilitate the adoption of validated biomarkers in clinical practice:

Engage Regulatory Bodies: For establishing the biomarkers new tests approval process supported by adequate evidence, cooperation with organizations like the FDA or EMA.

Develop Clinical Guidelines: The development of guidelines that seek to integrate biomarkers into existing clinical practice patterns will assist clinicians in the decision-making of patients.

Applying these particular strategies—multi-center studies, parallel with current diagnostic methods, comparative effectiveness, cutting-edge technologies, as well as, regulatory collaboration—the identification of bladder cancer biomarkers may be boosted. This will also put them into better service towards clinical practice to improve on early diagnosis and management of bladder cancer patients.

1. Comprehensive proteomics and platform validation of urinary biomarkers for bladder cancer diagnosis and staging.
2. Non-invasive Detection of Bladder Cancer by UBC Rapid Test, Ultrasonography and Cytology.

## Footnotes

1 B acillus C almette–G uerin.

2 Mucin antigen of the urinary bladder.
